# Childhood Cryptosporidiosis: A Case Report

**DOI:** 10.1155/2010/935625

**Published:** 2010-09-26

**Authors:** P. Agnamey, D. Djeddi, A. Diallo, A. Vanrenterghem, N. Brahimi, C. da Costa, A. Totet

**Affiliations:** ^1^Department of Medical Parasitology and Mycology, University Hospital, 80054 Amiens, France; ^2^Department of Paediatrics, University Hospital, 80054 Amiens, France

## Abstract

*Cryptosporidium* has emerged as an important cause of diarrheal illness worldwide, especially amongst young children and patients with infectious or iatrogenic immune deficiencies. The authors describe a case of mild cryptosporidiosis in a well-nourished, immunocompetent, one-year-old child. Rapid clinical and parasitological improvement was observed after a 3-day course of nitazoxanide.

## 1. Introduction

*Cryptosporidium,* a spore-forming protozoon, has been recognized as a human pathogen since 1976 [1]. The species most frequently involved in human infections are *Cryptosporidium hominis*, which primarily infects humans, and *Cryptosporidium parvum*, which infects humans and animals such as cattle, although infection with unusual species and genotypes occurs in both immunocompetent and immunocompromised populations [2]. In immunocompetent individuals, infection causes self-limited watery diarrhea. While, in patients with immune deficiencies, cryptosporidiosis may present as chronic or severe life-threatening diarrhea. In children, mainly those living in developing countries, cryptosporidiosis can lead to malnutrition and developmental delays [3]. Even asymptomatic infections are associated with growth deficits [4]. In industrialized countries, *Cryptosporidium* also has important public health implications. *Cryptosporidium spp.* are known to cause traveler's diarrhea [5] and they are also responsible for outbreaks of diarrhea, including a memorable outbreak in Milwaukee, Wisconsin, in 1993 during which nearly 403,000 people developed cryptosporidiosis due to contamination of drinking water [6]. These outbreaks highlight the medical importance of cryptosporidiosis. Antimotility drugs play a key role in the treatment of cryptosporidiosis in both immunocompetent and immunocompromised individuals, while the efficacy of antiparasitic drugs in cryptosporidiosis remains controversial, particularly in immunocompromised individuals [7]. A recent meta-analysis of trials of antiparasitic drugs in cryptosporidiosis revealed significant improvement of non-AIDS patients with nitazoxanide, but no clear evidence of efficacy for other antiparasitic drugs in cryptosporidiosis or for nitazoxanide in AIDS patients [8]. We describe the clinical and parasitological course of a child with cryptosporidiosis, who was treated successfully with nitazoxanide (Alinia, Romark Laboratories).

## 2. Case Report

A 16-month-old child was admitted to the pediatrics department of Amiens University hospital (France) for diarrheal syndrome associated with rhinitis. He was born in France to French parents living in a rural area who had never been outside of France. The child's father was a dairy farmer and his mother did not work on the farm. The child did not have any particular family history, but had a personal history of acute otitis media and rhinopharyngitis. His immune status was not investigated, but on the basis of this history of frequent rhinitis, a respiratory allergy was suspected and treated with antihistamine. 

The child presented to our institution with a six-day history of fever, rhinitis, vomiting, and profuse diarrhea. He experienced up to 7 episodes of nonbloody and nonglairy watery diarrhea per day. He was moderately dehydrated with very minor signs of dehydration such as weight loss, deep-set eyes, but no disorders of consciousness. On initial physical examination, the young patient was afebrile but presented tachycardia of 124 bpm, a respiratory rate of 18/min, and blood pressure of 82/52 mmHg. Oxygen saturation on room air was 99%. Chest, cardiac, and abdominal examinations were normal. On ear-nose-throat examination, the tympana could not be seen due to earwax and the throat was erythematous. One day after admission, after removal of earwax, ear examination revealed bilateral acute otitis media. The patient was placed on intravenous fluids and oral cefpodoxime was started while waiting for laboratory results. 

**Figure 1:**
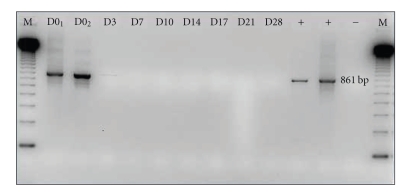
Amplification by nested PCR of *Cryptosporidium *DNA extracted from stool samples. The gel shows the amplification of a specific 861-bp fragment with specific primers [9]. Lane M: 123-bp DNA size marker ladder; lanes D0_1_ and D0_2_ (3 days later): samples collected before treatment; lanes D3–D28: samples collected after treatment; +: positive control; −: negative control.

The laboratory work-up revealed white blood cell count: 10,200 cells/mm^3^ with 46% neutrophils, 41% lymphocytes, 10% monocytes, 3% eosinophils; hemoglobin: 13.3 g/dL; platelets: 477,000/mm^3^. Basophilic lymphoid cells were observed. Blood biochemistry showed hyponatremia (130 mmol/L) and decreased alkaline reserve (19 mmol/L). Stool specimens were also sent for routine bacterial culture, rotavirus/adenovirus antigen, Giardia antigen, *Clostridium difficile* antigen, and all were negative. Routine stool examination for enteric parasites including direct saline wet mount examination and two concentration techniques: Bailenger's method and MIF (merthiolate iodine formaldehyde) with both a fixative and a stain was negative. *Cryptosporidium* antigen was detected in stool by the immunochromatographic method (RIDA QUICK Cryptosporidium, R-biopharm Diagnostic). Modified Ziehl-Nielsen staining of a stool smear showed several *Cryptosporidium* oocysts, up to 2,400 per gram of stool. Polymerase chain reaction-restriction fragment length polymorphism (PCR/RFLP) [9] identified the species as *Cryptosporidium parvum*. Three-day treatment (100 mg twice daily) with nitazoxanide suspension (Alinia, Romark Laboratories, FL, USA) was then initiated. The symptoms resolved on the day after the last dose of nitazoxanide. Oocysts were no longer detected in stool using the modified Ziehl-Nielsen stain and immunochromatographic methods, while PCR detection was still weakly positive. Eradication of oocyst excretion was observed on the day-7 stool sample and PCR detection was also negative (Figure 1). 

## 3. Discussion

*Cryptosporidium* infections obviously do not represent a major public health threat in developed countries, although recurrence of gastrointestinal symptoms due to *Cryptosporidium *is frequently reported, for example, in the Milwaukee outbreak in 1993 and in sporadic cases in Europe. In most sporadic cases, the source of infection is difficult to ascertain as many risk factors are commonly encountered in everyday life. Several factors facilitate transmission of *Cryptosporidium* and account for the propensity to cause large-scale outbreaks of diarrhea. (i) *Cryptosporidium *can infect many mammalian species. It is frequently identified in farm animals, particularly calves, and in domestic animals; (ii) the oocyst is very resistant and can conserve its infectivity in moist environments for a long time; (iii) *Cryptosporidium* genus is composed of a large number of species, several of which can infect humans; (iv) the infectious dose is very low, and infected individuals excrete large numbers of oocysts, up to 10^8^ in a single day [10]. *Cryptosporidium* oocysts also remain infectious after being shed and can cause self-infection. Public health surveys of cases must investigate all forms of exposure during the two weeks prior to onset of the illness in order to identify the source of contamination. In the present case, the child's father was a dairy farmer and the family lived 3 km from the farm. Two years prior to onset of the child's illness, the father reported cryptosporidium-related loss of calves in his livestock. The child had never been on the farm, and therefore had never had any contact with the animals. Parasitological stool examinations including specific methods for *Cryptosporidium* oocyst detection of other family members were negative, although the father reported occasional gastrointestinal disorders. *Cryptosporidium parvum* identified in the child and the documented presence of* Cryptosporidium* in the father's livestock are both in favour of these animals as the source of contamination, with transmission via the father. An environmental survey may have confirmed this hypothesis. As reported elsewhere [11, 12], this case report confirms the efficacy of nitazoxanide for the treatment of cryptosporidiosis in immunocompetent children. Rapid eradication of oocyst excretion was observed in this case. However, the role of the child's immune system in this rapid clearance of the parasite remains unknown. The negative result of *Cryptosporidium* antigen detection in stool observed immediately after the 3-day course of nitazoxanide is an interesting finding, suggesting that the rapid immunochromatographic method could be used for posttreatment stool tests instead of more time-consuming staining or PCR methods. 

This case highlights the need to consider spore-forming protozoa as potential causes of diarrhea in children. Parasite-related diarrhea in both immunocompetent adults and children is probably underestimated due to underdiagnosis. In contrast with bacterial and viral agents, parasites are less frequently considered by physicians as a potential cause of diarrhea. Clinical pathologists should therefore systematically perform screening for spore-forming protozoa in all patients with persistent or acute watery stools.

## References

[1] Nime FA, Burek JD, Page DL, Holscher MA, Yardley JH (1976). Acute enterocolitis in a human being infected with the protozoan Cryptosporidium. *Gastroenterology*.

[2] Cama VA, Bern C, Roberts J (2008). Cryptosporidium species and subtypes and clinical manifestations in children. *Emerging Infectious Diseases*.

[3] Guerrant DI, Moore SR, Lima AAM, Patrick PD, Schorling JB, Guerrant RL (1999). Association of early childhood diarrhea and cryptosporidiosis with impaired physical fitness and cognitive function four-seven years later in apoor urban community in northeast Brazil. *American Journal of Tropical Medicine and Hygiene*.

[4] Checkley W, Gilman RH, Epstein LD (1997). Asymptomatic and symptomatic cryptosporidiosis: their acute effect on weight gain in Peruvian children. *American Journal of Epidemiology*.

[5] Nair P, Mohamed JA, DuPont HL (2008). Epidemiology of cryptosporidiosis in north American travelers to Mexico. *American Journal of Tropical Medicine and Hygiene*.

[6] MacKenzie WR, Hoxie NJ, Proctor ME (1994). A massive outbreak in Milwaukee of cryptosporidium infection transmitted through the public water supply. *The New England Journal of Medicine*.

[7] Masur H, Kaplan JE, Holmes KK, Benson C Adult Prevention and Treatment of Opportunistic Infections Guidelines Working Group. Guidelines for prevention and treatment of opportunistic infections in HIV-infected adults and adolescents. http://aidsinfo.nih.gov/contentfiles/adult_oi.pdf.

[8] Abubakar I, Aliyu SH, Arumugam C, Hunter PR, Usman NK (2007). Prevention and treatment of cryptosporidiosis in immunocompromised patients. *Cochrane Database of Systematic Reviews*.

[9] Xiao L, Escalante L, Yang C (1999). Phylogenetic analysis of Cryptosporidium parasites based on the small- subunit rRNA gene locus. *Applied and Environmental Microbiology*.

[10] Chappell CL, Okhuysen PC (2002). Cryptosporidiosis. *Current Opinion in Infectious Diseases*.

[11] Rossignol J-F, Kabil SM, El-gohary Y, Younis AM (2006). Effect of nitazoxanide in diarrhea and enteritis caused by Cryptosporidium species. *Clinical Gastroenterology and Hepatology*.

[12] Smith HV, Corcoran GD (2004). New drugs and treatment for cryptosporidiosis. *Current Opinion in Infectious Diseases*.

